# Unpacking the dynamics of double stigma: how the HIV-TB co-epidemic alters TB stigma and its management among healthcare workers

**DOI:** 10.1186/s12879-020-4816-3

**Published:** 2020-02-06

**Authors:** Edwin Wouters, Nina Sommerland, Caroline Masquillier, Asta Rau, Michelle Engelbrecht, André Janse Van Rensburg, Gladys Kigozi, Koen Ponnet, Wim Van Damme

**Affiliations:** 1 Department of Sociology and Centre for Population, Family and Health, University of Antwerp, Sint-Jacobstraat 2, BE–2000 Antwerp, Belgium; 2 Centre for Health Systems Research and Development, University of the Free State, Bloemfontein, Republic of South Africa; 3 Centre for Rural Health, University of KwaZulu-Natal, Durban, South Africa; 4 Department of Communication Sciences, Media, Innovation and Communication Technologies, Ghent University, Ghent, Belgium; 5 International Health Policy, Department of Public Health, Institute of Tropical Medicine, Antwerp, Belgium

**Keywords:** Tuberculosis, HIV/AIDS, Co-epidemic, Stigma, double stigma, Health care workers, Structural equation modeling, South Africa

## Abstract

**Background:**

HIV and tuberculosis (TB) are intricably interlinked in South Africa. The social aspects of this co-epidemic remain relatively unexplored. More specifically, no research has quantitatively explored the double stigma associated with HIV and TB in this context, and more specifically the impact of the co-epidemic on [1] the stigmatisation of TB and [2] the TB stigma mangement strategy of covering (i.e. the use of TB as a cover for having HIV). The current study aims to address this research gap by disentangling the complex mechanisms related to HIV-TB stigma.

**Methods:**

Using Structural Equation Modelling (SEM), data of 882 health care workers (HCWs) in the Free State province, South Africa, are analysed to investigate the link between the stigmatization of HIV and TB and the stigma management by those affected. The current study focuses on health care workers (HCWs), as both TB and HIV have a severe impact on this professional group.

**Results:**

The results demonstrate that the perceived link between the epidemics is significantly associated with double HIV-TB stigmatization. Furthermore, the link between the illnesses and the double stigma are driving the stigmatization of TB. Finally, the link between HIV and TB as well as the stigmatization of both diseases by colleagues are associated with an increased use of covering as a stigma management strategy.

**Conclusions:**

This is the first quantitative study disentagling the mediating role of double stigma in the context of the co-epidemic as well as the impact of the co-epidemic on the social connotations of TB. The results stress the need for an integrated approach in the fight against HIV and TB recognizing the intertwined nature of the co-epidemic, not only in medical-clinical terms, but also in its social consequences.

**Trial registration:**

South African National Clinical Trials Register, registration ID: DOH-27-1115-5204. Prospectively registered on 26 August 2015.

## Background

HIV/AIDS and tuberculosis (TB) have merged into a deadly co-epidemic in South Africa. In absolute numbers, the country has the highest number of people living with HIV (7.0 million in 2016) [1–4]. In addition, it has one of the most severe TB epidemics in the world, with the highest incidence of TB (834 per 100,000 in 2015) and 19,613 reported cases of multi-drug resistant TB in 2015 [5]. Both epidemics are intricately intertwined: approximately 73% of TB cases are co-infected with HIV [6].

Ample research has addressed the clinical and health system challenges of the HIV and TB co-epidemic [7, 8], but – as Daftary (2012) rightfully pointed out – “the social aspects remain relatively unexplored” [9], especially the potential stigma associated with HIV and TB in a setting confronted with this dual epidemic. Evidence-based knowledge on the double stigma generated by the interlinked nature of both epidemics is urgently needed as both HIV and TB stigma have been individually extensively associated with non-disclosure [10–12], delayed health care [13–16], and ultimately worse (physical and psychological) health outcomes [10, 14, 17–21].

### TB/HIV stigma

Most studies on stigma, including those on HIV and TB stigma, are rooted in the work of Erving Goffman. He defined stigma as a discrediting social label that changes an individual’s self-image and disqualifies him or her from full social acceptance [22]. Goffman’s essay generated a plethora of research applying the concept to a wide range of circumstances, ranging from homosexuality to cancer, often with considerable variation in the concept’s definition, rendering it vulnerable to the critique that the stigma concept was too vaguely defined [23]. As a response, a more recent conceptualization by Link and Phelan (2001) narrows Goffman’s broad conception of stigma to the co-existence of its defining components, namely labeling, stereotyping, separation, status loss, and discrimination, all within a context in which power is exercised [24, 25].

The concept of stigma has been profusely applied to both HIV and TB. However, there has been little research on how the interlinked natures of the HIV and TB epidemics have impacted the stigmatization of both illnesses [9, 26–28]. Even fewer studies have investigated the occurrence of a “new” double stigma, despite Bond and Nyblade stating that “TB stigma can no longer be thought of, or addressed, separately from HIV stigma” and encouraging researchers to study and disentangle this double or compound stigma of HIV and TB [26].

A seminal article that responded to this call and addressed the construction and management of this double stigma was written by Daftary [9]. In this article, in-depth qualitative work examined the lived experience of co-infection with HIV and TB from the perspective of those affected in KwaZulu-Natal, South Africa. In the country, the co-epidemic renders TB not only symbolic, but also symptomatic of HIV [26, 27]: the symptoms associated with TB (e.g. weight loss) are also symptomatic of HIV, providing “impetus to the construction of overlapping dual stigmas” [9]. Evidence indicates that each raises different degrees of stigma, whereby TB appears as temporary and blameless while HIV is culpable and permanent. The stigmatization of HIV was thus greater than that of TB [9].

However, the confluence of both epidemics alters the situation: TB patients have emerged as a vulnerable group as the co-epidemic – in the current research setting, South Africa’s Free State province, 3 out of 4 TB cases with a known HIV status are HIV-positive [29] – renders them easy targets of HIV-related stigmatization. The negative affects normally associated with HIV are thus now – in the context of the co-epidemic – being transferred to TB. In a final step, Daftary [9] connected [1] the interlinked nature of both epidemics and [2] the resulting construction of overlapping – double – stigmas to [3] the social act of “covering” [9, 22]. “Covering” is a social act whereby people deflect attention away from a dominant stigmatizing attribute (in this case, HIV) by drawing attention to something more socially acceptable or less stigmatized (in this case, TB) [9, 22].

Despite the fact that several authors have asserted that, in the context of the co-epidemic, TB stigma cannot be studied separately from HIV stigma, there are – to the best of our knowledge – no quantitative studies attempting to disentangle this new compound or double stigma and its associations with TB stigma and TB stigma management. The current study aims to address this research gap by unraveling a range of concepts that are related to this double stigma. More specifically, we intend to build a model that represents social reality, linking the concepts raised by previous in-depth qualitative work, namely the link between HIV and TB, between the stigmatization of HIV and TB (double stigma) and the impact this has on the stigmatization TB and finally the potential reaction by those affected (covering). In other words, we expect that the epidemiological link between the 2 diseases results in a link between the stigmas (copying netagive connotations of HIV to TB), which in turn stimulates the stigmatization of TB and impacts on the reactions of those affected by the co-epidemic – i.e., by hiding a potential HIV-positive status with TB (covering).

### Health care workforce

The current study focuses on health care workers (HCWs) as the dual burden of TB and HIV has a severe impact on these professionals. Occupational exposure to TB constitutes a major health risk for HCWs [30, 31], especially in resource-constrained settings where large patient numbers and resulting overcrowded health facilities combined with poorly implemented infection control strategies render HCWs three times more likely to acquire TB than the general population [11, 32]. Consequently, TB is officially classified as an occupational hazard. The HIV epidemic equally affects the workforce because of the mutually reinforcing epidemiology of HIV and TB: estimates of the HIV prevalence among South African HCWs range from 11.5 to 20.0% [33].

In this context, workplace health services for HIV and TB are an essential part of any health system strengthening strategy [34]. Research has demonstrated that providing HIV and TB services to HCWs at work – in the occupational health unit (OHU) – is cost-effective and preferred by the majority of HCWs [35, 36]. Accordingly, a joint World Health Organization-International Labor Organization-UNAIDS policy document on the provision of TB and HIV prevention and care for HCWs explicitly recommends the on-site availability of such occupational health services for the entire workforce so that full access to HIV and TB prevention, treatment, care, and support can be attained for this vulnerable group [37].

However, a review paper demonstrated that HIV- and TB-related stigma and discrimination are “key barriers to both the delivery of quality health services by health providers and to their utilization by community members and health providers themselves” [38]. Stigmatization in the health care setting can thus have severe implications for HCWs and health facilities when HIV-positive/TB-infected HCWs delay or avoid care, causing increased morbidity and mortality and further strain on an overburdened health system [38]. It is thus important to include not only the respondent’s stigmatizing attitudes, but also the perceived stigmatizing attitudes of colleagues, as these may shape the views and behaviors of the participating HCWs.

This unique position of the health care workforce – bearing the burden of the co-epidemic on the front line of the health system while being at risk of contracting HIV and/or TB and consequent HIV and TB stigmatization – renders a quantitative study on double HIV-TB stigma among HCWs a clear research priority [11]. The current research thus aims to study (1) the interlinked nature of both epidemics and (2) the resulting construction of overlapping double stigmas (by the respondent and by colleagues) to (3) the stigmatization of TB (by the respondents and by colleagues) as well as (4) the resulting social act of “covering” in a sample of 882 HCWs in the Free State province of South Africa.

## Methods

The current study aims to explore the dynamics of double stigma and its impact on TB stigma (management) among HCWs by analyzing the baseline data of a cluster randomized controlled trial – the HIV and TB Stigma among Health Care Workers Study (HaTSaH study). The study was approved by the Ethics Committee of the Faculty of Health Science of the University of the Free State (ECUFS 55/2015) and the Ethical Committee for the Social Sciences and Humanities of the University of Antwerp (SHW-15-28-03).

The overarching HaTSaH study aims to (1) scientifically assess the extent and sources of HIV- and TB-related stigma among the health care workforce and (2) develop and test evidence-based stigma-reduction interventions. For this goal, a study sample of 882 HCWs – both clinical staff (446) (doctors and nurses) and non-clinical staff (436) (e.g. messengers, cleaners, and administrators) – working in 8 hospitals in the Free State was drawn from the health care workforce register. The questionnaires were in Afrikaans, Sesotho or English. After obtaining written informed consent from all participants, trained field workers provided the participants with the standard questionnaires that were completed in a self-administered process. Where literacy was low, the fieldworkers read the questions aloud, but they were answered independently.

### Measures

The current study aims to build a model that represents social reality, linking the concepts raised by previous in-depth qualitative work, namely:

- the link between HIV and TB (HIV-TB)
- the double stigma (HIV and TB) by the respondent (DS-R)
- the perceived double stigma (HIV and TB) by colleagues (DS-C)
- the stigma toward TB by the respondent (TBS-R)
- the perceived stigma toward TB by colleagues (TBS-C)
- the stigma management strategy of covering

The interlinked nature of the HIV and TB epidemics (HIV-TB) was measured by a scale with three items (“TB is a sign that someone has HIV”; “Someone with TB has probably also got HIV”; and “TB symptoms make HIV more noticeable”), each rated on a four point Likert scale indicating strong agreement, agreement, disagreement, or strong disagreement.

The interlinked nature of the stigmas as defined by the respondent (the Double Stigma by the Respondent, or DS-R) was measured by 1 item, namely “Someone who has TB should feel equally guilty about it as someone who has HIV,” – also rated on a four-point Likert scale.

The perceived interlinked nature of HIV and TB stigmatization by colleagues (perceived Double Stigma by Colleagues, DS-C) was measured by two items on a four-point Likert scale (“People are afraid of working together with someone who has TB because they think that the person also has HIV” and “People with TB tend to be treated badly because they may have HIV”).

The study also expects a significant impact on the perceived link between HIV and TB (HIV-TB) and between the stigmas (DS-R) on the stigmatizing attitudes and behavior toward TB. We therefore included a scale measuring the respondent’s stigmatizing attitudes toward TB (TBS-R, 2 items). In addition, we also expect a significant impact on the perceived link between HIV and TB and between HIV and TB stigmatization by colleagues (DS-C) on the perceived stigmatizing attitudes and behavior toward TB (TBS-C, 5 items) by colleagues. We consequently also included a scale measuring the colleagues’ stigmatizing attitudes toward TB (TBS-C, 2 items). The reliability and validity of these two scales measuring the respondent’s and colleagues’ stigma toward TB were reported in previous publications - where TBS-R, TBS-C were respectivelay labelled TB-RES and TB-OES [39–41].

The social act of “covering” was measured by two items, namely “If I was diagnosed with HIV then, in order to hide my HIV status, I would say that I have TB” and “If I was diagnosed with both TB and HIV I would only tell that I have TB,” each rated on a four point Likert scale. This social act is phrased in a conditional manner as we do not know the responding HCWs’ HIV or TB status.

As we expect that the TB-HIV co-epidemic has linked TB stigma to HIV stigma, we have to control for (1) respondent’s and (2) colleagues’ stigmatizing attitudes towards HIV. We included two scales measuring the respondent’s (HIVS-R, 4 items) and the collagues’ (HIVS-C, 4 items) stigmatizing attitudes toward HIV as we expect a significant impact of the perceived link between HIV and TB (HIV-TB) and between the stigmas (DS-R) on (1) the respondent’s and (2) the colleagues’ stigmatizing attitudes and behavior toward HIV.

In order to model social reality as closely as possible, a number of additional control variables are included in our analyses. The survey included a series of socio-demographic questions (age and sex). As we expect differences in various dependent variables between HCWs with medical training (nurses and doctors) and HCWs without any formal medical training (clerks, cleaners, administrators, etc.), this study will also include a measure of the professional category of the HCW (medical staff vs non-medical staff). In addition, this study includes the HIV- (10 items) and TB-related knowledge (10 items) of the health care workforce as the literature has repeatedly shown a link between knowledge and stigma [42, 43]. Finally, previous research [38, 44] clearly indicates that links exist between stigma and confidentiality. This survey thus included a question on confidentiality in the workplace: “In the last 2 years, have you personally ever witnessed an occupational health nurse in this hospital failing to keep confidentiality about the health status of another health care worker? (For example: gossiping about the health status of a health care worker).” Finally, the study also controlled for potential hospital effects arising from the cluster RCT design.

### Analytic strategy

**Fig. 1:**
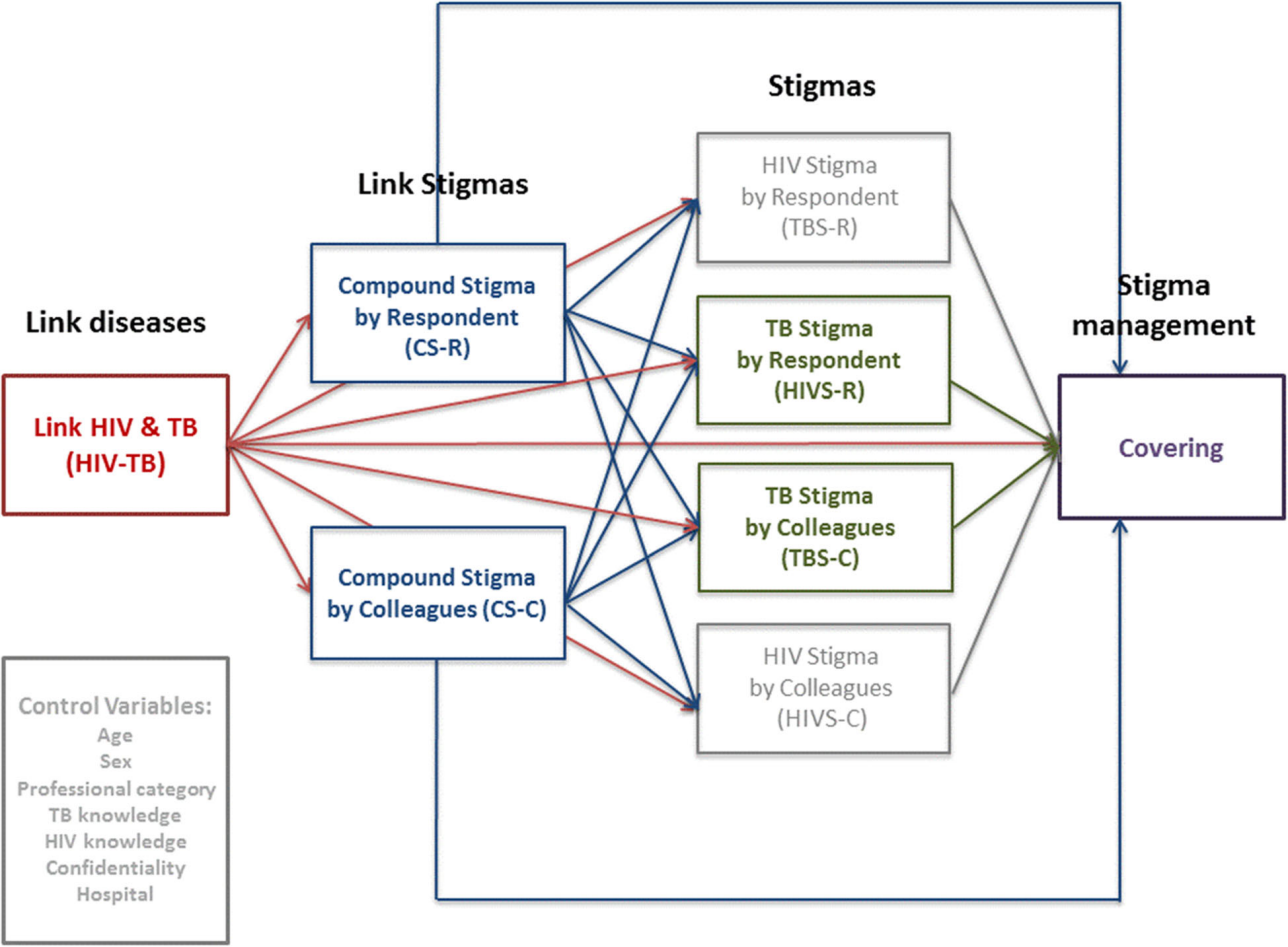
Conceptual model displaying the relationships between the epidemics, links between stigmas, TB stigma, and covering

Structural equation modeling (SEM) was used to measure the interrelationships between (1) the perceived interlinked nature of the two epidemics (HIV-TB), (2) the reported double stigmatization by the respondent (DS-R), and (3) the perceived double stigmatization by colleagues (DS-C) as well as the association of (1), (2), and (3) with the stigmatization by respondents of (4) TB (TBS-R) and the perceived stigmatization by colleagues of (5) TB (TBS-C). Finally, we also tested the impact of (1) to (5) on the intention to adopt the social act of (6) covering. All these relationships were controlled for age, sex, professional category, HIV knowledge, TB knowledge, confidentiality, and hospital effects. The conceptual model tested in this article is displayed in Fig. 1.

SEM is a combination of confirmatory factor analysis and multiple regression analyses. First, the measurement model specifies the relationships between the observed indicators (e.g., the items measuring the respondent’s double stigma) and the overarching latent variables (for example, the respondent’s double stigma). Second, the structural model specifies the relationships among the various observed and latent variables. Following the recommendations of Hu and Bentler (1999), two of the following three criteria had to be met for a satisfactory global model fit to be attained: CFI/TLI ≥ 0.95, RMSEA ≤0.06, and SRMR ≤0.08 [45]. All data analysis was performed using the statistical software package Mplus version 7.4. The MLM (maximum likelihood parameter estimates with standard errors and a mean-adjusted chi-squared test statistic that are robust to non-normality) method was used to fit the structural equation models to the dataset.

## Results

### Sample characteristics

The characteristics of our sample are depicted in Table 1.

**Table 1:** Sample descriptions

	Number	Percent
Gender		
Male	249	28.3%
Female	631	71.7%
Age (mean, SD)	875	43.62 (9.93)
Professional group		
Patient care	446	50.7%
Support staff	434	49.3%
HIV knowledge (mean, SD)	882	6.96 (1.65)
TB knowledge (mean, SD)	882	7.18 (1.52)
Breach in confidentiality	881	20.0%
Link between HIV & TB (HIV-TB)		
Item 1 (mean, SD)	882	3.21 (0.77)
Item 2 (mean, SD)	881	2.85 (0.87)
Item 3 (mean, SD)	879	2.52 (0.83)
Double stigma respondent (DS-R)		
Item 1 (mean, SD)	881	3.15 (0.74)
Double stigma by colleagues (DS-C)		
Item 1 (mean, SD)	880	3.03 (0.77)
Item 2 (mean, SD)	880	3.05 (0.76)
HIV stigma respondent (HIVS-R)		
Item 1 (mean, SD)	882	1.87 (0.78)
Item 2 (mean, SD)	882	1.65 (0.76)
Item 3 (mean, SD)	882	1.61 (0.70)
Item 4 (mean, SD)	882	1.50 (0.63)
TB stigma respondent (TBS-R)		
Item 1 (mean, SD)	882	1.70 (0.68)
Item 2 (mean, SD)	882	1.80 (0.67)
HIV stigma colleagues (HIVS-C)		
Item 1 (mean, SD)	881	1.99 (0.80)
Item 2 (mean, SD)	881	2.13 (0.83)
Item 3 (mean, SD)	880	1.99 (0.74)
Item 4 (mean, SD)	881	2.05 (0.81)
TB stigma colleagues (TBS-C)		
Item 1 (mean, SD)	879	1.98 (0.74)
Item 2 (mean, SD)	880	1.96 (0.72)
Item 3 (mean, SD)	879	2.12 (0.84)
Item 4 (mean, SD)	879	2.00 (0.77)
Item 5 (mean, SD)	880	2.91 (0.80)
Covering		
Item 1 (mean, SD)	882	3.06 (0.79)
Item 2 (mean, SD)	881	3.00 (0.80)

### Measurement model

Although SEM is a hybrid of factor analysis and path analysis, a two-step approach is recommended. Separate assessments of the measurement and structural models prevent the good fit of one model compensating for (and potentially masking) the poor fit of the other. The measurement model displays an excellent fit to the data (RMSEA = .046; CFI = .952; TLI = .941; and SRMR = .034) (Table 2). The reliability analyses display excellent consistency of the indicators in measuring the latent concepts, with Cronbach’s alphas ranging from .70 to .99. Only the 3-item scale measuring the interlinked nature of the HIV and TB epidemics (HIV-TB) displayed borderline consistency, with a Cronbach’s alpha of .66. The factor loadings of these three items were sufficiently high and ranged between .49 and .74. The two items measuring the perceived interlinked nature of HIV and TB stigmatization (double stigma) by colleagues (DS-C) also displayed sufficiently high factor loadings (λ = .81 and λ = .88). As reported in an earlier publication, the 4-item scale measuring the respondent’s stigmatizing attitudes toward HIV (HIVS-R) (λ ranging from .53 to .75) and the 2-item scale measuring the respondent’s stigmatizing attitudes toward TB (TBS-R) (λ = .71 and λ = .75) displayed sufficiently high loadings of the different items onto the factor [39]. Similarly, the 4-item scale measuring colleagues’ stigmatizing attitudes toward HIV (HIVS-C) (λ ranging from .69 to .72) and the 5-item scale measuring the colleagues’ stigmatizing attitudes toward TB (TBS-C) (λ ranging from .64 to .78) displayed sufficiently high loadings of the different items onto the factor [39]. Finally, both items measuring the social act of covering displayed high factor loadings (λ = .85 and λ = .70). Combined, these findings support the fit of the measurement model to the data as well as the reliability of these constructs and their indicators.

**Table 2:** Item analysis, goodness-of-fit, and reliability assessment of the measurement model (*n* = 874)

Scales	Standardized loadings (λ)	P
Link between HIV & TB (HIV-TB)		
Item 1	0.737	< 0.001
Item 2	0.663	< 0.001
Item 3	0.486	< 0.001
Double stigma by colleagues (DS-C)		
Item 1 (mean, SD)	0.814	< 0.001
Item 2 (mean, SD)	0.875	< 0.001
HIV stigma respondent (HIVS-R)		
Item 1 (mean, SD)	0.527	< 0.001
Item 2 (mean, SD)	0.734	< 0.001
Item 3 (mean, SD)	0.752	< 0.001
Item 4 (mean, SD)	0.742	< 0.001
TB stigma respondent (TBS-R)		
Item 1 (mean, SD)	0.711	< 0.001
Item 2 (mean, SD)	0.754	< 0.001
HIV stigma colleagues (HIVS-C)		
Item 1 (mean, SD)	0.720	< 0.001
Item 2 (mean, SD)	0.686	< 0.001
Item 3 (mean, SD)	0.699	< 0.001
Item 4 (mean, SD)	0.689	< 0.001
TB stigma colleagues (TBS-C)		
Item 1 (mean, SD)	0.728	< 0.001
Item 2 (mean, SD)	0.782	< 0.001
Item 3 (mean, SD)	0.685	< 0.001
Item 4 (mean, SD)	0.643	< 0.001
Item 5 (mean, SD)	0.733	< 0.001
Covering		
Item 1 (mean, SD)	0.851	< 0.001
Item 2 (mean, SD)	0.704	< 0.001
Goodness of fit		
RMSEA	0.046	
CFI	0.952	
TLI	0.941	
SRMR	0.034	

### Structural model

The RMSEA (0.039) indicates a close fit of the overall model with reasonable errors of approximation in the population. Combined with the other goodness-of-fit statistics (CFI = 0.929; TLI = 0.905; and SRMR = 0.038), we can conclude that the structural model displays an acceptable fit to the data (Table 3).

**Table 3:** Standardized Mplus coefficients (β) and model summary of the structural model (*n* = 862)

	Link between stigmas		Separate stigmas				Management
	DS-R	DS-C	TBS-R	TBS-C	HIVS-R	HIVS-C	Covering
HIV-TB	0.497***	0.425***	0.238***	0.062	0.178**	−0.024	0.235***
DS-R			0.152***	0.059	0.078	0.014	0.038
DS-C			0.430***	0.611***	−0.040	0.571***	0.085
TBS-R							0.402***
TBS-C							− 0.234*
*Control variables:*							
HIVS-R							− 0.005
HIVS-C							0.352***
Age	− 0.000	− 0.004	0.085	− 0.036	0.050	0.000	0.051
Sex	−0.003	−0.051	0.068	− 0.018	0.048	− 0.009	0.072*
Professional category	−0.190	0.017	0.064	0.016	0.058	0.033	0.023
TB knowledge	0.004	−0.128**	− 0.105**	−0.013	− 0.099*	0.014	0.056
HIV knowledge	−0.051	0.004	−0.019	−0.010	− 0.095*	0.044	− 0.042
Confidentiality	0.069*	0.061	0.078*	0.148***	0.016	0.179***	−0.011
Hospital (Ref = 1)							
Hospital 2	−0.054	−0.041	− 0.034	−0.066*	0.001	−0.007	0.009
Hospital 3	−0.002	0.005	−0.089	−0.009	− 0.023	−0.041	− 0.024
Hospital 4	−0.028	0.009	−0.017	− 0.008	−0.085	0.012	0.013
Hospital 5	−0.014	0.015	−0.001	0.001	0.002	0.050	0.013
Hospital 6	0.019	−0.027	− 0.012	0.014	− 0.055	−0.007	− 0.046
Hospital 7	0.028	−0.029	−0.111**	− 0.033	−0.083*	− 0.023	0.079*
Hospital 8	0.035	0.004	−0.099**	−0.027	− 0.005	−0.019	0.084*
Goodness of fit							
RMSEA	0.039			TLI	0.905		
CFI	0.929			SRMR	0.038		

*P < .05; **P < .01; ***P < .001

The first section of the model assessed the association between (1) the interlinked nature of the epidemics (HIV-TB) and both (2) the interlinked nature of the stigmas (double stigma) (DS-R) as defined by the respondent and (3) the perceived double stigmatization by colleagues (DS-C). The reported link between both epidemics was significantly and positively associated with the reported link between HIV stigma and TB stigma (β = .497; *P* < .000). Respondents who reported a strong link between HIV and TB also reported a stronger link between the negative attributes and resulting double stigmatization of both illnesses. The reported link between both epidemics was also significantly and positively associated with the perceived double stigmatization by colleagues (β = .425; *P* < .000). In other words, health care workers who reported that having TB is a sign of having HIV were more likely to report that co-workers stigmatize people with TB because of HIV-related reasons. Conversely, TB knowledge was negatively associated with the perceived double stigmatization by colleagues (β = −.128; *P* < .005): health care workers with higher levels of TB knowledge were less likely to perceive a transfer of the stigmatizing attitudes toward HIV to TB among their colleagues. Finally, witnessing a breach in confidentiality at the sick bay was positively associated with the perceived link between HIV stigma and TB stigma by the respondent (β = .069; *P* < .05). None of the other control variables was significantly linked to these two 2 outcomes.

The second section explored whether (1) the link between the epidemics (HIV-TB), (2) the reported link between the stigmas (DS-R), and (3) the perceived link between the stigmas by colleagues (DS-C) are associated with the stigmatization of TB – in other words, how the link between the illnesses and the link between their stigmas is reflected in the stigmatization of TB. We assessed the association of these 3 aspects of the co-epidemic with (1) the stigmatization of TB by the respondent (TBS-R) and (2) the perceived stigmatization of TB by colleagues (TBS-C).

First, the link between the epidemics (β = .238; *P* < .001), the double stigma (as reported by the respondent) (β = .152; *P* < .005), and the perceived double stigma by colleagues (β = .430; P < .001) were significantly associated with the stigmatizing attitudes and behaviors of the responding HCW toward TB: it is clear that the stigma toward TB is strongly associated with the co-epidemic and its resulting double stigma. Respondents who reported that TB is a sign of having HIV, who linked the two stigmas, and who perceived that colleagues linked the 2 stigmas were significantly more likely to stigmatize TB. Age (β = .085; *P* < .05), less TB knowledge (β = −.105; *P* < .01), and witnessing a breach in confidentiality (β = .078; *P* < .05) were also significantly associated with TB stigma: older HCWs with lower levels of knowledge of TB were more likely to report stigmatizing attitudes and behaviors toward TB. None of the other control variables – apart from a few hospital effects – were significantly linked to TB stigma.

Second, the perceived link between the stigmatization toward HIV and TB by colleagues was significantly associated with the corresponding TB stigma type, namely the perceived stigmatization toward TB by colleagues (β = .611; *P* < .001). There was thus a very strong association between the perceived double stigma and the separate TB stigma. Witnessing a breach in confidentiality at the sick bay was also significantly associated with the perceived stigmatization toward TB (β = .148; *P* < .001) by colleagues. None of the other control variables was significantly linked to TB stigmatization by colleagues.

When looking at the relationships with the stigmatization towards HIV (by the respondent and by colleagues) – one of our most important control variables – we observe that the reported link between HIV and TB (HIV-TB) was positively associated with HIV stigmatization by the interviewed HCW (HIVS-R) (β = .178; *P* < .005). HCWs who reported that TB is a sign of having HIV were more likely to report stigmatizing attitudes and behaviors toward HIV. Having more HIV (β = −.095; *P* < .05) and TB (β = −.099; *P* < .05) knowledge was negatively associated with stigmatizing attitudes toward HIV: HCWs who knew more about HIV and TB were less likely to stigmatize HIV-positive colleagues. In accordance with the findings on TB stigma, the perceived link between the stigmatization toward HIV and TB by colleagues was significantly associated with the corresponding HIV stigma types, namely (1) the perceived stigmatization toward HIV by colleagues (β = .571; *P* < .001). Witnessing a breach in confidentiality at the sick bay was also significantly associated with the perceived stigmatization toward HIV (β = .179; P < .001) by colleagues.

The structural model also includes the correlates of the social act of covering. Covering was significantly associated with (1) sex, (2) the reported link between HIV and TB (HIV-TB), (3) the stigmatization of TB (TBS-R), (4) the perceived stigmatization of TB by colleagues (TBS-C), and (5) the perceived stigmatization of HIV by colleagues (HIVS-C).

Being female (β = .106; *P* < .05) weakly but significantly increased the odds of using TB as an excuse for having HIV when being confronted by these illnesses. HCWs who recognized having TB as a sign of having HIV (link between both epidemics) were significantly more likely (β = .235; *P* < .001) to employ TB as a cover for HIV when they become infected HIV (or both HIV and TB). Thirdly, there was a strong link (β = .351; *P* < .005) between the self-reported stigmatization of TB and the reported likelihood of using TB as cover for having HIV: HCWs who displayed higher levels of TB stigma were significantly more likely to resort to covering when they were HIV-positive. There was a strong positive link between the stigmatization toward TB and the likelihood of adopting the social act of covering (β = .402; *P* < .001): against expectations, HCWs with stigmatizing attitudes toward TB were significantly more likely to cover an HIV-positive diagnosis with the label of having TB. The perceived TB stigmatizing behavior by colleagues was, as expected, negatively associated with covering (β = −.233; *P* < .05): if HCWs responded that their colleagues stigmatized TB more, they were significantly less likely to employ TB as a cover for HIV. Correspondingly the perceived HIV stigmatizing behavior by colleagues was positively associated with covering (β = .350; P < .001): if HCWs responded that their fellow HCWs stigmatized HIV more, they were significantly more likely to use TB as a cover for HIV.

## Discussion

Sub-Saharan Africa at large and South Africa in particular is confronted with a devastating HIV/TB-co-epidemic. Little is known, however, of the creation and dynamics of double stigma in the context of these intertwined epidemics and the impact of this double stigma on the social connotations of TB. The current study addressed this research gap by explicitly exploring how (1) the interlinked nature of HIV and TB and (2) the resulting double stigmatization of HIV-TB (by the respondent and by colleagues) have changed the stigmatization TB and the resulting stigma management strategy of (4) covering.

As previously reported in the qualitative work of Daftary (2012) and Bond and Nyblade (2006), the interwovenness of HIV and TB, reflecting the bio-medical reality of HIV-TB co-infection, has resulted in the creation of a new form of compound or double stigma [9, 26]. The results of the analysis clearly indicate that the link between both illnesses is reflected in an additional layer of stigmatization, that of the transfer of stigmatizing attitudes and behaviors previously linked to HIV and TB. It was clearly demonstrated that the perceived link between the epidemics was reflected in the creation of a double stigma, reflected in both (1) a self-reported link between both stigmas and (2) a perceived linking of both stigmas by fellow HCWs. The study thus provides quantitative evidence of the linking of both illnesses and resulting stigmas previously reported in exploratory qualitative studies [9, 26].

The current study went a step further by subsequently exploring the link between this double stigma and the stigmatization of TB. As expected, the link between the epidemics and the associated double stigma has resulted in the increased stigmatization of TB. To the best of our knowledge, this is the first quantitative study to demonstrate the empirical link between the interwovenness of HIV and TB and the stigmatization of TB with the associated compound or double stigma as an important mediating variable. The results offer an evidence-based explanation of why TB-associated stigma has declined in low HIV-prevalence communities but resurged in high-burden settings during the past decade [9, 46].

Finally, this study explored the drivers of the stigma management strategy of covering, whereby people living with HIV attempt to avoid or minimize discrimination by using a lesser stigmatized illness, TB, as a cover for HIV. Our findings indicate that the perceived interlinked nature of the two epidemics clearly drives this stigma management strategy. In line with previous research [9], HCWs who recognize the link between HIV and TB are more likely to employ covering as a way to minimize HIV-related discrimination. It is thus evident that the co-epidemic and its repercussions for the stigmatization of HIV and TB impact the behavior of those affected.

In contrast with expectations, stigmatizing attitudes against TB increased the likelihood of employing the stigma management strategy of covering, meaning that one would employ TB as a cover for HIV. However, this can be explained by the fact that, as indicated above, a large part of the variability in TB stigma can be ascribed to the transfer of discriminatory attitudes against HIV to individuals with TB. This was also shown in the qualitative work of Mavhu et al. (2010) and Ngamvithayapong et al. (2000) [47, 48]. Those who stigmatize TB are thus those who recognize the link between both epidemics and potentially see covering as a vital or effective strategy to avoid HIV stigmatization. Conversely, it is also possible that we have a case of reversed causality: it is possible that those HCWs viewing sharing a (fictitious) TB diagnosis as a potential strategy to cover for HIV, are transfering stigmatizing attitudes toward HIV to TB, thereby displaying increased levels of TB stigma. The fact that TB stigma for many respondents seems almost synonymous with the double stigma as the stigmatization of TB is largely attributable to its link with HIV renders it virtually impossible to singularize the relationship between TB stigma and covering in abstraction of the link with HIV.

In line with expectations, the perceived stigmatizing attitudes of colleagues toward TB were strongly associated with the likelihood of employing covering as a strategy to deal with a positive HIV diagnosis. The stronger the dominant stigma associated with HIV and the weaker the stigma associated with TB, the more likely HCWs were to resist discrimination by using TB as a cover for HIV. Our results demonstrate that the stigmatizing attitudes toward HIV by colleagues clearly push HCWs toward potentially deflecting “attention away from a dominant stigmatizing attribute (that is, HIV) by drawing attention to something more socially acceptable (that is, TB)” [9]. Our results also support the statements of Mbonu et al. (2009) who concluded in their systematic study that “people prefer to claim that they are bewitched or have (normal) tuberculosis rather than accept that they have HIV/AIDS” [49]. Our study is the first large-scale quantitative study to confirm these exploratory qualitative findings.

This study’s strengths include: (1) its theoretical foundation in stigma literature, (2) its large-scale quantitative design, (3) the availability of well-developed scales to measure different types of stigma (TB, HIV, and double stigma), and (4) the availability of information on an understudied population, namely HCWs active in a vulnerable health system burdened by the HIV/TB co-epidemic. To the best of our knowledge, this is the first study to quantitatively disentangle the complex interrelationships between the interlinked nature of the epidemics, the interlinked nature of the stigmas, the distinct HIV- and TB-related stigmas, and the resulting stigma management strategy (covering). We empirically tested and confirmed the theory-building findings of previous in-depth qualitative studies on this topic.

This study had several limitations. First, it focused on the stigma management strategy of covering, which has emerged as one of the most relevant stigma management strategies in the context of the co-epidemic. Other stigma management strategies (for example, “othering,” the symbolic distancing from persons affected by HIV) were not examined; future studies should incorporate these alternative stigma management strategies. Second, although cross-lagged regression would have been more appropriate, the study was limited to cross-sectional analyses as the follow-up data of the cluster RCT were not yet available. We were therefore unable to make any attributions of causal influence among the different independent variables and stigma outcomes. Third, this study collected data on a random sample of HCWs active in the 8 selected hospitals, irrespective of their HIV status or TB history, thus gathering data on their stigma management strategies in the hypothetical case of a TB/HIV co-infection. Fourth, this study did not explore stigma related to drug-resistant TB (MDR-TB), which is known to have distinct and more severe manifestations disturbing the examined relationships between HIV and TB stigma and the resulting management strategy. Given these shortcomings, future longitudinal, large-scale studies collecting data on a random sample of co-infected people with a specific focus on MDR-TB are needed to confirm and potentially expand knowledge on the demonstrated interrelationships.

## Conclusions

The results of this study have both theoretical and practical relevance. From a theoretical perspective, this is the first study to use a large-scale dataset to empirically disentangle the interrelationships between HIV, TB, HIV stigma, TB stigma, and double stigma. The results demonstrate that, as hypothesized by previous qualitative work, the co-epidemic (and especially the link with the discredited HIV status) is driving the stigmatization toward TB. Consequently, the findings extend our understanding of the social ramifications of the co-epidemic by empirically supporting the theorization of double stigma and the resulting management of the two interlinked illness-related identities in the context of the devastating HIV-TB co-epidemic.

These insights help to inform appropriate responses from the health care system: from a practical and policy perspective, the results urge policymakers to strengthen integrated measures, as the interlinked nature of the epidemics and the associated stigmas demonstrate that vertical programs inevitably ignore a significant part of the reality of an HIV or TB diagnosis. The results urge policymakers to merge the often-contrasting cultures of HIV care, a patient-centered, individualized approach, and TB care characterized by a traditional public health approach [8, 50, 51]. The proposed integrated approach should recognize the intertwined nature of the HIV-TB co-epidemic, not only in medical-clinical terms, but also in its social consequences. The established rise in TB stigma caused by the perceived links between HIV and TB and the resulting double stigma however also urge health policy makers to not blindly intergrate HIV and TB care as this may increase TB stigma levels. The current push towards integration [52, 53] thus needs to be complemented with interventions that recognize and take into account the interlinked nature of the epidemics, which cannot be separated in a context where 3 out of every 4 TB cases are HIV-positive [27, 50]. Our findings and the associated overarching HaTSaH study testing an intervention to address HIV and TB stigma among HCWs aligns with the ongoing START study by Howard et al. [54] that explicitly aimed to evaluate the effectiveness of a combination intervention package to improve ART uptake and adherence during TB treatment in Lesotho. More research is needed to translate the knowledge of the social ramifications of the co-epidemic into evidence-based interventions that can optimally combat the co-epidemic and not only reduce the risk of transmission (and the resulting burden of disease), but also create a health-enabling environment in which HCWs affected by HIV and/or TB are optimally treated and supported [11, 55].

## Data Availability

The data that support the findings of this study are available from the HaTSaH team but restrictions apply to the availability of these data, which were used under license for the current study, and so are not publicly available. In order to get access to the data on which the present study is based, permission from the HaTSaH study is necessary. The Data and Publication Committee of the HaTSah study evaluates all applications for access to data, and upon approval of application, an agreement is made between the HaTSaH study team and the project manager of the project in question. Questions regarding access to data may be directed towards edwin.wouters@uantwerpen.be.

## Abbreviations

CFI: Comparative Fit Index
DR-R: The double stigma (HIV and TB) by the respondent
DS-C: The perceived double stigma (HIV and TB) by colleagues
HaTSaH: studyHIV and TB Stigma among Health Care Workers Study
HCW: Health care worker
HIV-TB: The link between HIV and TB
MLM: Maximum likelihood parameter estimates with standard errors and a mean-adjusted chi-square test statistic
OHU: Occupational health unit
RMSEA: Root Mean Square Error of Approximation
SEM: Structural equation modeling
SRMR: Standardized root mean squared residual
TB: Tuberculosis
TBS-C: The perceived stigma toward TB by colleagues
TBS-R: The stigma toward TB by the respondent
TLI: Tucker-Lewis Index
